# Self-reported tooth loss and cognitive function: Data from the Hispanic Established Populations for Epidemiologic Studies of the Elderly (Hispanic EPESE)

**Published:** 2013-09-30

**Authors:** Carlos A. Reyes-Ortiz, John S Luque, Charlotta K Eriksson, Libia Soto

**Affiliations:** 1 Oakwood Hospital & Medical Center, Department of Internal Medicine, Division of Geriatrics, Dearborn, Michigan, Division of Geriatrics, Department of Internal Medicine, Oakwood Hospital and Medical Center, Dearborn, Michigan.; 2 Jiann-Ping Hsu College of Public Health, Georgia Southern University, Statesboro, Georgia; 3 School of Odontology, Universidad del Valle, Cali, Colombia.

**Keywords:** Tooth loss, cognitive function, Mexican Americans, older, longitudinal analyses

## Abstract

**Objective:**

To explore the association between tooth loss and cognitive functioning among persons 65 years and older.

**Methods:**

Data from the Hispanic Established Populations for Epidemiologic Studies of the Elderly (Wave 1: 1993-1994, n= 3,032; Wave 2: 1995-1996, n= 2,424; and Wave 3: 1998-1999, n= 1,967). The dependent variables were the scores from the total Mini-Mental State Examination (MMSE: score 0-30) and its global domains (memory: score 0-6; and no-memory: score 0-24). Independent variables included the number of teeth (0-12 vs. 13-32), socio-demographic characteristics, last dental office visit, medical conditions, depressive symptoms, and functional limitations which were tested for associations with the dependent variables.

**Results:**

In bivariate analyses, participants with fewer teeth (0-12) tended to have significantly lower mean scores for memory, no-memory, and total MMSE when compared to those with more teeth (13-32), both at baseline and at follow-up. In fully adjusted longitudinal-mixed models, participants with fewer teeth had a greater decline in total MMSE through five years of follow-up with a decrease of 0.12 fewer points each year (SE ± 0.05, *p* <0.01), when compared to those with more teeth.

**Conclusion:**

Having fewer teeth was associated with greater cognitive decline over time.

## Abstract (es)

**Objetivo:**

Explorar la asociación entre pérdida de dientes y función cognoscitiva en las personas de 65 años o más.

**Métodos:**

Datos del estudio EPESE (Poblaciones Establecidas para los Estudios Epidemiológicos de los Ancianos) Hispano (Encuesta 1: 1993-1994, n= 3,032; Encuesta 2: 1995-1996, n= 2,424; y Encuesta 3: 1998-1999, n= 1,967). Las variables independientes fueron el Examen MiniMental total (MMSE, 0-30 de puntaje) y sus dominios globales (memoria: 0-6 de puntaje; y no-memoria: 0-24 de puntaje). Variables independientes fueron el número de dientes presentes en la boca (0-12 vs. 13-32), características sociodemográficas, última visita al odontólogo, condiciones médicas, síntomas depresivos, y limitaciones funcionales a las cuales se les hicieron pruebas de asociación con las variables dependientes.

**Resultados:**

En análisis bivariados, los participantes con pocos dientes (0-12) tenían promedios de puntajes mas bajos en memoria, no-memoria y el MMSE total, comparado con los participantes con mas dientes (13-32) en la encuesta inicial y al seguimiento. En modelos mixtos longitudinales ajustados por todas las variables, los participantes con menos dientes tenían un declive mayor en el MMSE total a través de los cinco años de seguimiento, con una disminución de 0.12 puntos cada año (ES ± 0.05, *p* <0.01), comparado con quienes tenían mas dientes.

**Conclusión:**

La presencia de pocos dientes estuvo asociada con un mayor declive cognoscitivo a través del tiempo.

## Introduction

Several studies have shown a relationship between cognitive impairment and poor oral health. Tooth loss, as a result of chronic, subclinical inflammation and chronic periodontitis has been linked to cognitive decline^1^ ^-^ ^5^. Grabe *et al*. ^1^, reported that having fewer teeth was associated with lower Mini-Mental State Examination (MMSE)^6^ scores, especially among older women. In another study, Noble *et al*. ^2^, reported an association between *Poryphyromonas gingivalis* IgG levels (a pathogen causally associated with chronic periodontitis) and poor delay verbal memory or serial subtractions scores. In addition, using NHANES data (1999-2002), Wu *et al*. ^3^, reported that lower Digit Symbol Substitution Test^7^ scores (assessing psychomotor performance, attention, response speed, visuomotor coordination and incidental memory) were associated with decayed teeth, missing teeth, and chronic periodontitis in older adults. 

The Hispanic population aged 65 and older reached 2.7 million in 2008 and is projected to grow to over 17 million by 2050. By 2019, the Hispanic older population is projected to be the largest ethnic minority in this age group category^8^. Rates of cognitive impairment appear to be higher among older Hispanics than rates found in other older non-Hispanic populations^9^. Little is known about the relationship between cognitive impairment and oral health among older Hispanic populations in the United States. The objective of this study was to explore the association between tooth loss (as a proxy of chronic periodontitis)^1^ and cognitive function, using longitudinal data from the Hispanic Established Populations for Epidemiologic Studies of the Elderly (EPESE)^10^. 

## Materials and Methods

### Design and Sample

The Hispanic EPESE is a prospective longitudinal panel study of non-institutionalized Mexican Americans aged 65 and older residing in Texas, California, New Mexico, Colorado, and Arizona. It included an initial cross-sectional baseline survey with annual follow-up contacts of all participants and continual surveillance of mortality and use of hospitals and nursing homes. The primary purpose of the study was to provide estimates of the prevalence of key physical health conditions, mental health conditions and functional impairments in older Mexican Americans and to compare those estimates over time in the same individuals. 

The sampling included multi-stage, stratified, area probability procedures and was developed by listing counties in the five above Southwestern states by the number of Mexican American residents in descending order that were needed to cover 90% of all Mexican Americans. Census tracts and enumeration districts in the above counties were subsequently listed by the number of older Mexican Americans. Three hundred census tracts were selected with probabilities proportional to the number of Mexican American elderly as primary sampling units (PSU's), which provided clusters for door-to-door screening. A block group was selected in each census tract using a systematic random selection process (multiplying a random integer by the number of households). In some cases a second or third block was added to ensure a minimum of 400 households in each sampling unit. 

In-home interviews were conducted with 3,050 subjects of Mexican origin aged 65 and over and identified by U.S. 1990 Census procedures. This sample was calculated to be generalizable to 500,000 older Mexican Americans and was sufficiently large to provide stable estimates of most health characteristics of interest at follow-up. The response rate was 83%, which was equal to or better than that of the other EPESE surveys (East Boston, New Haven, the Piedmont region of North Carolina and rural Iowa). 

The subjects were interviewed and examined in their own homes by trained interviewers. Interviewers were trained by Harris Interactive, Inc. staff, medical personnel, and by Hispanic EPESE investigators and staff who provided education on blood pressure measurement, performance-based assessments of physical functioning, height and weight measures, waist and hip measures, vision assessment, medication use, and other measures^10^. The interviews were conducted in Spanish or English, depending on the participant's preference. Interviewers were fully proficient in both Spanish and English. A pretest of the baseline questionnaire and physical measurements was completed with a sample of persons 65 years of age and older from Bexar and Galveston Counties in Texas. The objectives of the pretest were: (1) to examine the length, flow, and content of the interview and to identify problems and inconsistencies with questions; (2) to evaluate the assessment techniques for blood pressure measurement and performance-based assessment of physical functioning, including training methods and procedures; and (3) to assess the validity of the Spanish-language version of the questionnaire. The results of the pretest provided information for reducing the length of the questionnaire and recommendations for revising the format, clarifying questions, and making minor changes in wording.

The study was approved by the University of Texas Medical Branch Institutional Review Board and written consents were obtained from each participant. The results for this article were obtained by secondary data analyses of the Hispanic EPESE; personal identifiers were previously deleted. The present study used baseline data (1993-1994, n= 3,032), and data obtained from follow-up at two years (Wave 2: 1995-1996, n= 2,424), and five years (Wave 3: 1998-1999, n= 1,967).

### Measures

The dependent variables included participants' total MMSE^6^ scores (0-30), memory scores (0-6) and no-memory scores (0-24)^11^ at baseline (Wave 1) and at follow-up (Waves 2 and 3). The two global domains, memory and no-memory scores, were validated in a previous study^11^. The memory domain comes from the sum of two sub-domains: working memory (or immediate/registration memory: score 0-3) and delay memory (or recall memory: score 0-3). The no-memory domain comes from the sum of three sub-domains: orientation (score 0-10), attention (score 0-5), and language (score 0-9)^11^. The English and Spanish versions of the MMSE were adopted from the Diagnostic Interview Scale (DIS) and have been validated among Hispanic populations in prior community surveys^12^. The Spanish version was translated from the English version without any modification of the items. 

The main independent variable, number of teeth, was assessed at baseline. Participants were asked, "How many of your own teeth do you still have?" A card with the following options was shown: "1) 0 (or none, edentulous); 2) 1-12 (or 1/4); 3) 13-19 (or 1/2); 4) 20-27 (or 3/4); and, 5) 28-32 (or all)?" The number of teeth was dichotomized as less than half (0-12) and half or more (13-32). 

At baseline, participants were also asked when they last visited a dental office (less than one year, 1-2 years, or > 2 years ago). Other variables were included since previous studies using data from the Hispanic EPESE found them to be associated with MMSE or other covariates^11^ ^,^ ^13^ ^-^ ^17^. The socio-demographic variables examined were age (in years), gender, preferred language at interview (Spanish or English), and education (in years). 

Corrected bilateral near vision acuity was measured by having participants hold cards at least seven inches from their eyes and asking them to read the numbers^14^. Each card had seven-digit telephone numbers of three different font sizes: 7, 10, and 23 points. Participants who could only read the 10, 23 point fonts or were unable to read the 23 point font size were considered to have near vision impairment. Participants who could read the 7 point font size were considered to have adequate near vision. 

Medical conditions were assessed with a series of questions asking the respondents if they had ever been told by a doctor that they had hypertension, diabetes, heart attack, or a stroke. Depressive symptoms were measured by the Center for Epidemiologic Studies Depression Scale (CES-D)^18^. This scale consists of 20 items in which participants are asked whether they have experienced certain feelings or symptoms in the past week. Response items are scored on a 4-point scale (0-3), with an overall range of 0 to 60, where higher scores indicate increased depressive symptoms. The conventional cut-off point of 16 was used to classify respondents as "depressed" (or as having clinically relevant depressive symptoms), or "not depressed"^18^. 

Functional status was assessed using seven items from a modified version of the Katz Activities of Daily Living (ADL) scale^19^. ADLs included walking across a small room, bathing, grooming, dressing, eating, transferring from a bed to a chair, and using the toilet. Participants were asked if they could perform the activities without help, if they needed help, or if they were completely unable to do so. ADL score (range 0-7) was used as a continuous variable. 

### Statistical analyses

**Figure 1:**
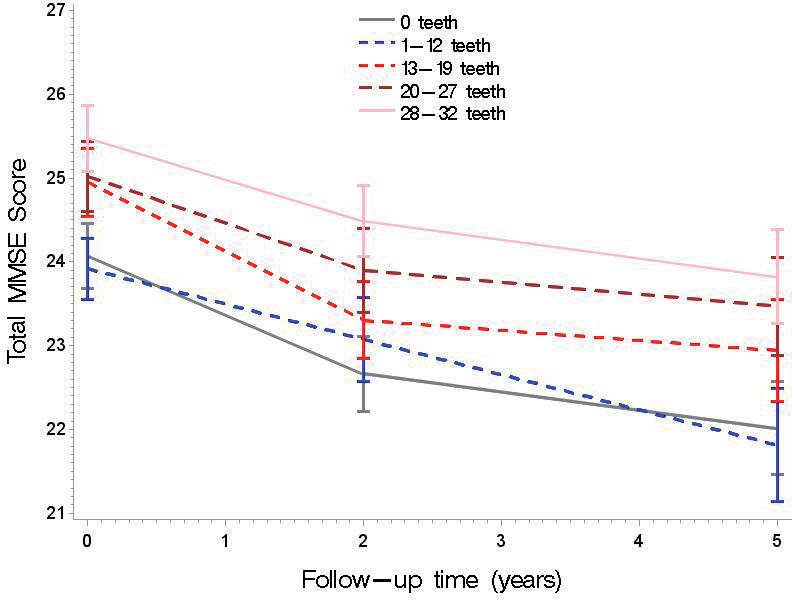
Unadjusted means (95% confidence intervals, vertical lines) and slope of decline (oblique lines) in total Mini-Mental State Examination (MMSE) scores by number of teeth with five categories measured at baseline, 2, and 5 years of follow-up.

**Figure 2:**
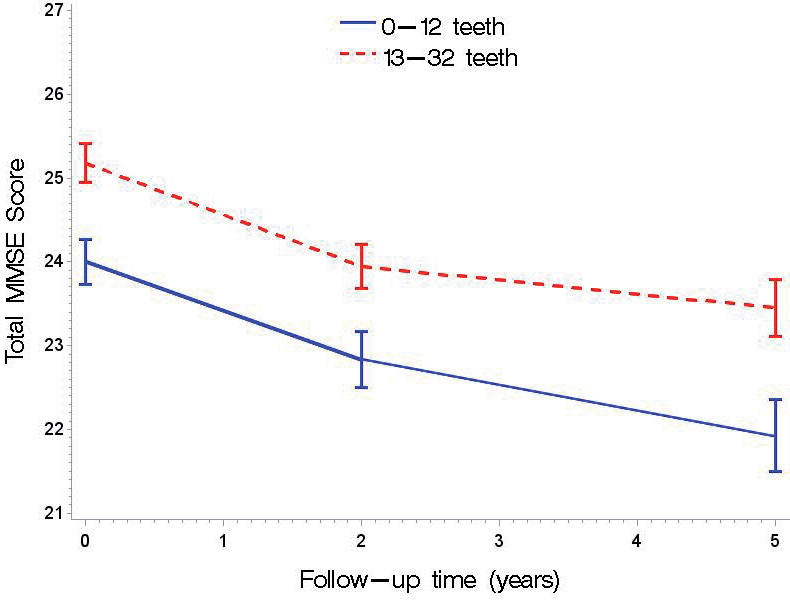
Unadjusted means (95% confidence intervals, vertical lines) and slope of decline (oblique lines) in total Mini-Mental State Examination (MMSE) scores by the dichotomized number of teeth as measured at baseline, 2, and 5 years of follow-up.

All analyses were performed using the SAS(r) System for Windows, version 9.3 (SAS Institute, Inc., Cary, NC), and the significance level was set at *p* <0.05, for two-tailed tests. To describe the study population across the categories of the number of teeth, we used descriptive statistics (% or means ± SD). To determine associations between the categories of number of teeth with other variables, we used the Mantel-Haenszel Chi-square test for categorical variables or the Kruskal-Wallis non-parametric ANOVA test for continuous variables (Table 1). To test whether number of teeth (either by five categories or dichotomized) was associated with cognitive function (total MMSE) over time, we fitted unadjusted general linear mixed models (Figs 1 and 2). We conducted multivariate longitudinal analyses (MIXED procedure) for each MMSE global domain (memory in Table 2; no-memory in Table 3) and total MMSE score (Table 4) as a function of the number of teeth (dichotomized). We performed cross-sectional analyses (without the number of teeth/time interaction term) and longitudinal analyses (with the number of teeth/time interaction term), including the variable time using two models.

In Model 1, the number of teeth was adjusted for age, gender, education, language at interview, and last dental office visit. In Model 2, the number of teeth was additionally adjusted for near vision impairment, hypertension, diabetes, heart attack, stroke, depressive symptoms, and ADL limitations. To predict MMSE and its domains over time, dental health measures and socio-demographic variables were used only at baseline. Near vision impairment was also used only at baseline. Variables used as time-dependent covariates were language spoken at interview, medical conditions, depressive symptoms, and ADL limitations. 

## Results

Twenty-three percent of participants had last visited a dental office in less than one year, 17% within 1-2 years, and 60% more than 2 years ago. For data analysis, the variable for the last time visiting a dentist was dichotomized as 0-2 or >2 years, as was done in a previous study^12^. Table 1 shows the study population characteristics at baseline, according to the number of teeth. Fewer teeth were associated with older age, female gender, lower education, and a longer time lapse since visiting a dentist. Fewer teeth were also associated with near vision impairment, history of stroke, diabetes, heart attack, depressive symptomatology, and ADL limitations. Additionally, participants with fewer teeth tended to have lower mean scores for memory, no-memory and total MMSE, when compared to those with more teeth.

**Table 1:**
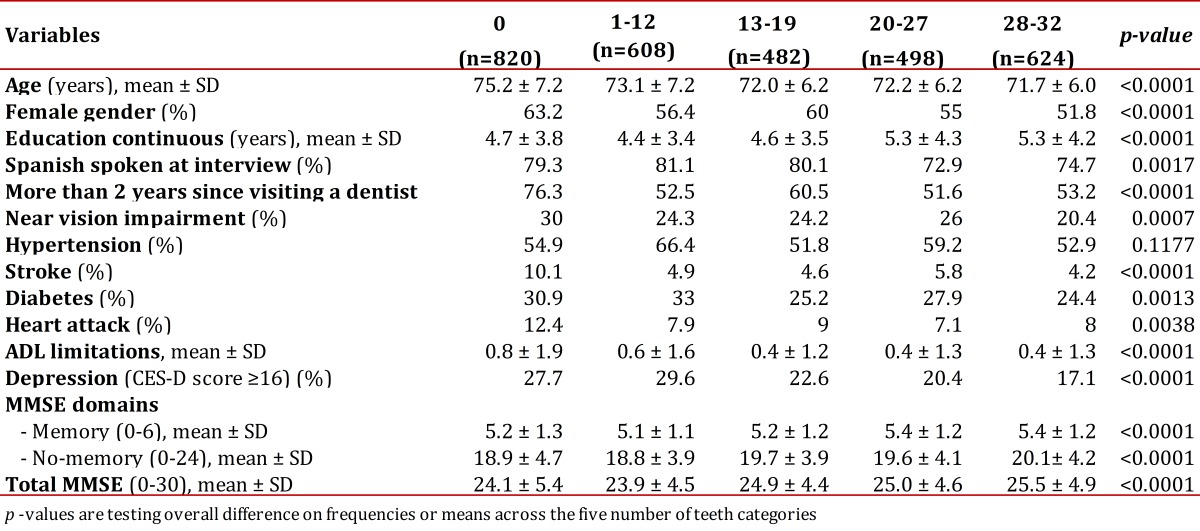
Study population at baseline (Wave 1) according to number of teeth categories.

For further analyses, we dichotomized the number of teeth (0-12 vs. 13-32) because there was no statistically significant difference on MMSE scores at follow-up between group 1 (edentulous or 0 teeth) and group 2 (1-12 teeth). However, the other groups (3= 13-19 teeth; 4= 20-27 teeth; and 5= 28-32 teeth) had statistically significant lower MMSE scores when compared to groups 1 and 2 (*p* <0.05). 

Figures 1 and 2 present unadjusted means in total MMSE scores (with 95% confidence intervals) by categories of number of teeth (five categories and dichotomized, respectively) as measured at baseline and at two and five years follow-up. Participants with fewer teeth tended to have significantly lower scores of MMSE (vertical lines) compared to those with more teeth at baseline and at follow-up. This association was stronger when the number of teeth was dichotomized (according to the non-overlapping confidence intervals). On the other hand, the slopes of decline (oblique lines) were deeper for participants with fewer teeth when compared to participants with more teeth, especially when the number of teeth was dichotomized (for 0-12 teeth its estimate is -0.16 SE± 0.05; the reference is 13-32 teeth; *p*= 0.0009). Therefore, we chose to dichotomize the variable for the number of teeth in the multivariate analyses. 

Table 2 shows the multivariate mixed models predicting memory scores over the five-year follow-up as a function of number of teeth and other characteristics. The variable time is testing the slope of the overall memory scores over time; a negative sign preceding the estimate indicates a decline. For example, for Model 1, there was an overall drop of 0.10 points (SE ± 0.01, *p* <0.0001) for memory scores each year. Also, those with fewer teeth (0-12), compared to participants with more teeth (13-32), had significantly lower memory mean scores (estimate= -0.07, SE ± 0.03, *p* <0.05). However, in Model 2, these results were no longer significant. Longitudinal results (with the interaction term of teeth/time) indicated that the interaction term between the number of teeth and time represented the longitudinal effect of the baseline measure of number of teeth on the annual rate of decline in performance of memory. However, the results were not significant for Model 3 or Model 4. 

**Table 2:**
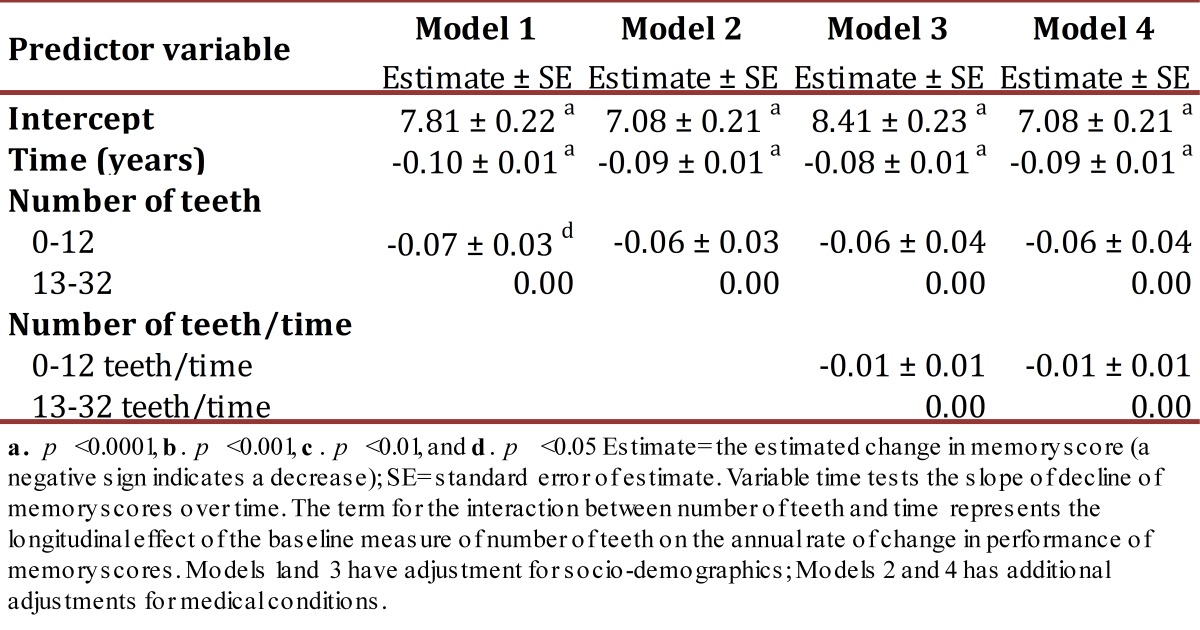
Multivariate analyses (Mixed models) of scores for memory domain (score 0-6) as a function of number of teeth and other variables, from Wave 1 through Wave 3

Estimate= the estimated change in memory score (a negative sign indicates a decrease); SE= standard error of estimate. Variable time tests the slope of decline of memory scores over time. The term for the interaction between number of teeth and time represents the longitudinal effect of the baseline measure of number of teeth on the annual rate of change in performance of memory scores. Models 1and 3 have adjustment for socio-demographics; Models 2 and 4 has additional adjustments for medical conditions.

Table 3 shows the adjusted mixed models predicting no-memory scores over the 5-year follow-up as a function of number of teeth and other characteristics. Model 1 showed an overall drop of 0.38 points (SE ± 0.02, *p* <0.0001) for no-memory scores each year. Also, compared to participants with more teeth (13-32), those with fewer teeth (0-12) had significantly lower no-memory mean scores (estimate= -0.47, SE ± 0.13, *p* <0.001). In Model 2, the results are still significant (estimate= -0.29, SE ± 0.11, *p* <0.05). For the longitudinal results, those with a lower number of teeth (0-12) compared to participants with a higher number of teeth (13-32), had a greater decline in no-memory scores through five years of follow-up. There was a drop of 0.12 points more each year (SE ± 0.04, *p* <0.01) in both Model 3 and Model 4 . 

**Table 3.**
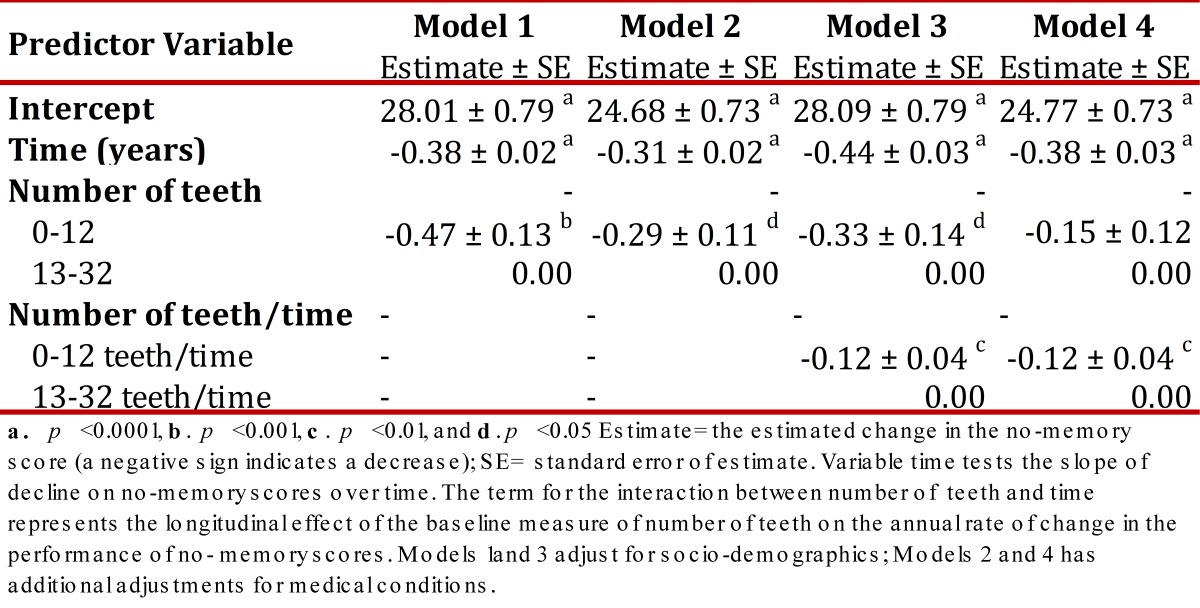
Multivariate analyses (Mixed models) of scores for the no-memory domain (score 0-24) as a function of the number of teeth and other variables from Wave 1 through Wave 3

Estimate= the estimated change in the no-memory score (a negative sign indicates a decrease); SE= standard error of estimate. Variable time tests the slope of decline on no-memory scores over time. The term for the interaction between number of teeth and time represents the longitudinal effect of the baseline measure of number of teeth on the annual rate of change in the performance of no-memory scores. Models 1and 3 adjust for socio-demographics; Models 2 and 4 has additional adjustments for medical conditions.

Table 4 shows the adjusted mixed models predicting total MMSE scores over the five-year follow-up as a function of number of teeth and other characteristics. In Model 1 there was an overall drop of 0.48 points (SE ± 0.02, *p* <0.0001) for total MMSE scores each year. Also, those with fewer teeth (0-12) compared to participants with more teeth (13-32), had significantly lower total MMSE mean scores (estimate= -0.56, SE ± 0.16, *p* <0.001). In Model 2, the results remained significant (estimate= -0.36, SE ± 0.13, *p* <0.01). For the longitudinal results, those with fewer teeth (0-12) compared to participants with more teeth (13-32), had a greater decline in total MMSE scores through five years of follow-up. There was a drop of 0.13 fewer points each year (SE ± 0.05, *p* <0.01) in Model 3, and 0.12 fewer points each year in Model 4 (SE ± 0.05, *p* <0.01). 

**Table 4:**
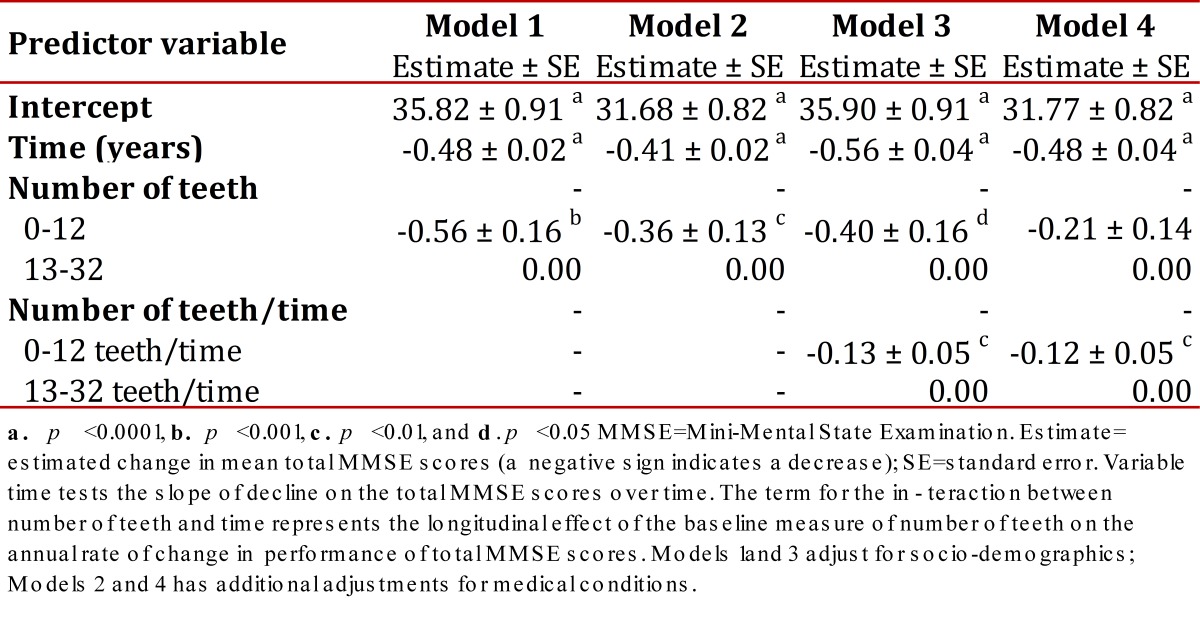
Multivariate analyses (Mixed models) of scores for total MMSE (score 0-30) as a function of number of teeth and other variables from Wave 1 through Wave 3

MMSE=Mini-Mental State Examination. Estimate= estimated change in mean total MMSE scores (a negative sign indicates a decrease); SE=standard error. Variable time tests the slope of decline on the total MMSE scores over time. The term for the interaction between number of teeth and time represents the longitudinal effect of the baseline measure of number of teeth on the annual rate of change in performance of total MMSE scores. Models 1and 3 adjust for socio-demographics; Models 2 and 4 has additional adjustments for medical conditions. 

## Discussion

We examined the relationship between the number of teeth and cognitive functioning among Mexican Americans aged 65 and older. We found that having fewer teeth was associated with greater cognitive decline over time. This was particularly true when predicting the no-memory domain or the total score of the MMSE. In the longitudinal analyses these associations were significant even after adjustment for potential confounders, such as socio-demographic variables, near vision impairment, co-morbidities, depressive symptoms, and functional status. 

Other studies have found associations between the number of teeth and cognitive function^1^ ^-^ ^6^. In a longitudinal study, Kaye *et al.* ^4^, found that rates of tooth loss and periodontal disease progression predicted subsequent decline in cognitive function, as measured by lower MMSE scores among older men. Also, in the Fujiwara-Kyo study, older participants with the lowest remaining number of teeth (0-10) were more likely to have mild memory impairment and low MMSE score (≤23) when compared to those with more teeth (22-32)^6^. The association between cognitive decline and tooth loss might be bidirectional as the Wu *et al*.^3^ study found that reduced cognitive performance predicted tooth loss. In a recent study, a higher gingival index, a measure of gingival inflammation, was associated with cognitive impairment at follow-up for older participants^20^. 

On the other hand, chronic periodontitis has been suggested as part of the pathogenesis of Alzheimer's disease (AD), the most common cause of dementia. Indeed, destructive forms of periodontal disease that ultimately result in tooth loss have been linked to the etiology of AD. Although hypothetical, it has been proposed that periodontitis-derived cytokines could reach the brain by both systemic and neural pathways and amplify brain cytokine pools^21^. For example, in a case-control and co-twin control study (monozygotic pairs in which one was diagnosed with dementia and the other was determined to be non-demented, thus controlling for genetic influences) that used data from the Swedish Twin Registry, Gatz *et al*.^22^, found that history of tooth loss before age 35 was a significant risk factor for having Alzheimer's disease. 

By contrast, other studies have not found an independent association between number of teeth and cognitive function. For example, Mathews *et al*.^23^, used data from a stroke study covering a population 45 years of age and older from eight U.S. states. They reported a bivariate association between cognitive function and self-reported tooth loss, where participants with the loss of six or more teeth had poorer cognitive function (learning or delay recall scores) when compared to those who had not lost teeth. However, after adjusting for socioeconomic status, the association disappeared. In another study, using data from NHANES III, Stewart et al.^24^ found that tooth loss was no longer associated with the story recall score after adjusting for socioeconomic factors among participants 70 years of age and older. 

This study has some limitations. First, the number of teeth was self-reported by the participants and not confirmed by a dentist. This results in a potential over/underestimation of its true number. However, in previous studies with the same database, the number of teeth was highly correlated with the use of dentures and other variables usually associated with poor oral health^13^ ^,^ ^17^. Second, the number of teeth may be a marker of overall health related to dental trauma, or it may reflect other confounding factors that we did not measure. Third, one cannot demonstrate causality from an observational study. 

Nevertheless, an important contribution of this study is the potential benefit of oral health on cognitive functioning in this particular population, older Mexican Americans. One strength of this study was its longitudinal analysis. We found only two previous studies that used longitudinal or prospective data^4^ ^,^ ^20^. Also, the findings in this study remained significant after controlling for time-dependent covariates that were not considered in the analyses of other previous studies and are likely to affect cognitive functioning. 

## Conclusion

Our results over five years of follow-up show that fewer teeth predict cognitive decline in older Mexican Americans that are independent of socio-demographic variables and health factors such as visual impairment, medical conditions, and functional impairment. Further studies using more objective measures of tooth loss or periodontitis are needed to continue exploring the relationship between poor oral health and cognitive function. 
